# Integrating agentic artificial intelligence into pasture-based dairy systems: Applications, governance, and future directions

**DOI:** 10.3168/jdsc.2025-0961

**Published:** 2026-03-27

**Authors:** Callum Eastwood, Nick Lim, Rachel Durie, Brian Dela Rue, Albert Bifet, Charlotte Reed

**Affiliations:** 1 Research and Science, DairyNZ, Hamilton 3240, New Zealand; 2 AI Institute, Waikato University, Hamilton 3216, New Zealand; 3 Perrin Ag Consultants, Rotorua 3010, New Zealand

## Abstract

Digitalization and artificial intelligence (AI) provide opportunities for improved management of pasture-based dairy systems. Agentic AI, where autonomous systems can perceive and act independently of humans, are potentially transformative. This mini-review explores the potential use of agentic AI to address key challenges in pasture-based dairy systems. Applications include autonomous grazing animal health prediction, environmental modeling, and virtual assistants. Agentic AI can integrate multiple data sources to support real-time, farm-specific decisions. It also presents opportunities for employee training, enhanced advisory services, and digital twin modeling. However, deployment of agentic AI introduces governance, ethical, and socio-technical considerations. Issues of data ownership, transparency, explainability, and trust must be addressed. The experiential and tacit knowledge of farmers must be integrated into AI systems through hybrid intelligence (human and AI). Oversight models ranging from Human-in-the-Loop to Human-in-Command are necessary to ensure safe and responsible use. Future research should focus not only on technical AI development, but farmer-centered design, robust assurance frameworks, and inclusive and responsible innovation ecosystems that align technological progress with dairy sector, civil society, values, and needs.

## Highlights

• Agentic AI presents an opportunity for dairy farm management.
• Complexity in pasture-based dairies may suit agentic capabilities.
• Use of multiple data types could enable precision farming.
• Farmer-centered design is essential for benefits, such as reduced cognitive load, to be realized.
• Governance frameworks must ensure trust, transparency, and ethical use of agentic AI tools.

## Graphical Abstract

The integration of agentic artificial intelligence (AI) into pasture-based dairy systems presents opportunities and wider considerations for farmers, researchers, and policymakers. Agentic AI can autonomously process multiple data streams to generate actionable decisions within the farm system, including grazing management, animal health, environmental sustainability, workforce productivity, and advisory services. The development of hybrid intelligence between farmers and AI by combining their strengths could lead to better farm systems outcomes than either could achieve alone. Wider considerations in the development of agentic AI systems involve AI governance, transparency and explainability, skill development, and the development of useful and trustworthy technology through farmer-centered design. Responsible innovation principles need to underpin the development of agentic systems.

**Figure:**
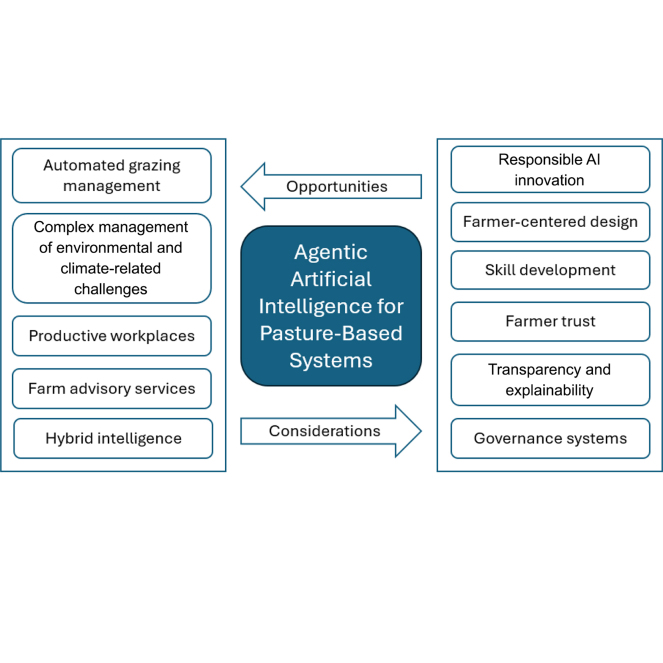

Dairy farming businesses rely on timely decision-making, skilled people, and effective advisory networks (Nettle et al., 2015; Hogan et al., 2023; Kaur et al., 2023). Seasonal pasture-based dairy systems face challenges such as matching pasture supply with herd feed demands and block calving. They also require management of peak workloads over calving and attracting skilled people capable of managing complex biological systems within highly variable weather conditions. Given these operational pressures, tools that streamline and improve decision-making have the potential to be transformative.

Digitalization and data analytics have been suggested as methods to enhance precision and accuracy of dairy farm management (Aquilani et al., 2022; Edwards et al., 2024; Selvaggi et al., 2024). However, the uptake of digital tools on pasture-based dairy farms has been variable. More widely adopted technologies include on-animal sensors (Edwards et al., 2026) and use of automation and sensors at milking (Dela Rue et al., 2019; Palma-Molina et al., 2023b; Prendergast et al., 2024). Less adopted technologies include robotic milking, virtual fencing, and pasture management data and tools (Eastwood et al., 2020; Palma-Molina et al., 2023a). Barriers to digitalization include technical constraints (e.g. data quality, interoperability), farm system factors (e.g. cost of integration and skills required), and innovation system barriers related to identity, equity, and societal expectations (Eastwood et al., 2023; Kelly et al., 2025). Although these barriers constrain digitalization on farms, advances in artificial intelligence (**AI**) for agricultural research and technology development have advanced rapidly.

Innovation in AI since the 2010s has advanced the ability to aggregate and analyze large datasets (Lamanna et al., 2025). Large language models, generative AI (**GenAI**), and agentic AI can address several barriers to dairy farm digitalization and change how farmers and their advisory networks interact with dairy knowledge systems. Artificial intelligence is already transforming activities across the dairy supply chain, from farm to processing and distribution (Khanashyam et al., 2025). This review summarizes current knowledge on the use of agentic AI and its potential application to address challenges and provide opportunities in pasture-based dairy systems. First, we describe and define agentic AI, then propose how its use might interact with aspects of pasture-based systems, and finally we outline broader considerations for the future development and integration of agentic AI on dairy farms.

Although AI has been in development since the 1950s, large language model (**LLM**)-based tools captured global attention after the launch of ChatGPT in 2022 (Lamanna et al., 2025). Subsequently, powerful “agentic” versions of AI are being developed. Agentic AI systems (agents) analyze data, make decisions, and act with minimal human intervention (Hughes et al., 2025). Multiple agents can validate outputs and operate in complex and multimodal environments (where data comes from many sources; Acharya et al., 2025; Li et al., 2025). The spectrum of autonomy these agents can have range from lower-order reactive, rule-based systems to higher-order fully autonomous and potentially self-aware agents, where higher levels require less human oversight (Table 1; Bornet et al., 2025; Nisa et al., 2026). Examples of rule-based agentic AI are use of imagery and climate data to trigger smart irrigation and frost alarm activities (Tariq et al., 2025). Traditional decision-making tools, software, and automation used in dairy farming (e.g., herd-management software) need significant human input and generally focus on farm system components. Well-designed and trained agent-based systems can provide decision-making support that better accounts for the farm system complexity.

**Table 1:** Artificial intelligence agent progression framework with potential example use cases in pasture-based dairy systems (adapted from Bornet et al., 2025)

Level of agency	Descriptor (adapted from Bornet et al., 2025)	Examples for potential use in pasture-based farming
0	Manual/no agency No decision-making initiative No ability to act without human control	Spreadsheet-based feed models with inbuilt algorithms. Farm systems models. Manual coding of invoices in accounting systems.
1	Rules-based systems Assists humans but does not take control Predefined triggers If-then rules based	Decision-tree-based approach to pasture management or fertilizer decisions. Autosorting of cows by a rules-based agent with control over sort gate. Variable rate irrigation based on rules-driven decisions with soil moisture and climate inputs.
2	Intelligent automation Able to take some independent action Uses basic machine learning	Late-lactation dry-off decisions based on predicted feed availability and costs, reproductive performance goals, and longer-term genetic improvement goals. System that interprets invoices and automatically codes. Accuracy is improved over time, learning from past decisions.
3	Agentic workflows Reasoning capabilities Can operate without human oversight Short-term learning based on feedback	Farm-specific GPT tools where AI is making its own inferences and solving user problems. Self-driving tractors for silage production. Autonomous pasture assessment robots. AI-powered maintenance scheduler monitors machinery and automatically orders parts or books services when predictive models identify maintenance requirements.
4	High autonomy Broad autonomy over a wide range of tasks. Multimodal capability Autonomous task planning and execution	Adjustment of feed allocation based on real-time data such as pasture availability, weather patterns, body condition score, and access to virtual fencing technology. Digital farm consultant learns from historical farm data and market signals to autonomously recommend and execute seasonal strategies.
5	Full autonomy/agency Advanced reasoning, problem solving Environmental awareness	Fully autonomous robotic systems making decisions based on farm-based data. Fully autonomous farm management system operating the entire farm (e.g., scheduling milking, managing and purchasing feed, hedging milk contracts, coordinating staff and resources) and is optimized for long-term profitability and sustainability.

Pasture-based dairy production occurs worldwide (Wilkinson et al., 2020), particularly countries such as Ireland, New Zealand, Australia, Uruguay, Argentina, and Chile (Moscovici Joubran et al., 2021). Farmers in pasture-based dairy systems must carefully manage feed variability due to reliance on grazed pasture. This creates unique challenges in matching feed demand with pasture supply, which can vary daily due to dynamic climate-biological interactions, and coupled with grazing management decisions can affect pasture quality and quantity (Verkerk, 2003; Wilkinson et al., 2020). Pasture growth seasonality and seasonal calving can require a concentrated breeding period that requires a focus on reproductive efficiency. Grazing cattle are exposed to variable health risks such as lameness, mastitis, facial eczema, and thermal stress, resulting in peak workloads and transition cow health risks during calving (Verkerk, 2003; Eastwood et al., 2025a). Seasonal pressures are complicated by a changing and more variable climate, increasing decision uncertainty (Garcia et al., 2021; Nettle et al., 2025a).

Issues include changing growth patterns within and between seasons, occurrence of pest and disease issues, extreme weather events requiring adaptive management of drought, wet soils, and diverse forage systems (Eastwood et al., 2025b). Agentic AI can simplify decision-making, for example, embedding agentic AI in grazing management software could connect weather forecasts, pasture data, on-farm sensing (Edwards et al., 2024; Pinna et al., 2024), and farm-specific decision rules for forage management. This could enable autonomous grazing decisions with minimal human-in-the-loop intervention. There is also potential to integrate external factors, such as live imported feed prices, into an agentic feed tool to support proactive feed procurement. Uncertainty in climate-biological systems and nuances of individual farm contexts (Beukes et al., 2019; Leddin et al., 2023) provide challenges for current human decision-making processes, and AI systems will also need to operate within, and may be more capable in dealing with, this uncertainty. Grazing decision-making often relies on tacit knowledge learned experientially by farmers (Hall et al., 2017; Eastwood et al., 2020), which is often not digitized or accessible to AI systems. Further, this tacit knowledge may be difficult for farmers to articulate to LLM or other agentic AI systems.

The management of animals within pasture systems, and the need to make vital decisions during high workload periods, presents another immediate opportunity for agentic AI, largely due to advances in animal-centric digitalization. The adoption of on-animal sensors (wearables) has increased rapidly in recent years (Palma-Molina et al., 2023b; Edwards et al., 2026). Recent studies have highlighted this animal-level data as highly useful for predictive analytics and agent-model collaboration where AI agents can access and query existing computer models (Eckhardt et al., 2025; Liu et al., 2025). A vital component of agentic AI use with animals, especially in higher level autonomous systems, is that animal welfare is maintained as a priority (Dawkins, 2025). Advances in machine learning (**ML**), AI, and integration of sensor data have been predicted to improve illness and heat stress prevention and detection, treatment of disease, identification of novel phenotypes (Verdon et al., 2025), or mitigation of heat stress (Hendriks et al., 2025). The AI systems could also adapt to longer-term climate variability. Using edge AI (AI algorithms on local devices or Internet of Things devices), agentic systems could be embedded in on-farm technologies to make immediate autonomous decisions related to aspects such as milking schedules, animal health management, and heat stress mitigation, particularly when combined with virtual herding technologies (Langworthy et al., 2021; Ghavi Hossein-Zadeh, 2025).

Environmental challenges have become more pressing for pasture-based dairy systems, particularly around efficiency of nitrogen use (Wilkinson et al., 2020; Lakhanpal et al., 2025) and greenhouse gas emissions (Garnett and Eckard, 2024; Marmont et al., 2024). There are complex system interactions when addressing environmental issues that can lead to unintended outcomes (Eastwood et al., 2025b). This presents an opportunity for higher-order agentic AI and its ability to interact with farm systems models (Liu et al., 2025) for uses such as autonomous fertilizer decisions and application. Farmers, advisors, and scientists could use these agents to interrogate “digital twin” models with a range of scenarios to explore benefits and consequences of strategic farm systems change (Lee et al., 2025).

In addition to peak workload over calving, pasture-based dairy farm workplaces require working outside in variable weather and working within small farm teams where a range of skills are required (Santhanam-Martin et al., 2021; Hogan et al., 2023). Studies have also suggested that workplace productivity has not improved in the past decade in several pasture-based dairy sectors (Beca, 2021). In these systems, grazing-related tasks and herding cattle require more labor input compared with housed systems (Hogan et al., 2022). The concept of an agent-enabled “virtual dairy farm assistant” is gaining traction (Ferreira and Dórea, 2025; Nisa et al., 2026), and since 2020 GenAI or rules-based AI agents (Table 1) have begun to appear in the agriculture sector (Liu et al., 2025). A virtual assistant has the potential to be used for information access, automation of key farm decisions, autonomous reconciliation of supplies and procurement. Such systems have also been proposed in domains such as the medical profession (Moor et al., 2023; Zou and Topol, 2025). Agentic assistants could improve productivity through faster decision-making, autonomous control of certain workplace tasks, saving time on office and compliance tasks, and reducing the hours required to run the farm business in a partial solution to issues with attracting and retaining farm staff (Dedieu et al., 2022; Hogan et al., 2024).

In the development of agentic systems, broader questions about implications on dairy workplaces need to be addressed. One issue is how automated decision-making affects farmer agency, if decision-making is automated through AI. An AI future could enhance or diminish the role of the farmer and have impacts on the attractiveness of the role. Studies in other sectors have examined the impact on employee identity seen in other studies (Qin et al., 2025). The perceptions and impact of AI will depend on whether AI is viewed as augmenting their roles, rather than automating them (Eastwood et al., 2021; Ryan, 2025). Agentic AI may also affect labor and there may be a reduced need for skilled labor due to virtual assistants, and farm roles and skills may shift to a greater focus on oversight of farm operations.

Key aspects in pasture-based systems can include the management of grazing, block calving and mating, soil nutrients and effluent, maintenance of components such as fences, troughs, and effluent systems, calf collection, and winter cropping. Attracting and retaining people with this wide range of skills is becoming increasingly difficult (Eastwood et al., 2019b; Nettle et al., 2025b) and industry training systems are struggling to stay relevant to changing expectations of new generations (Cosby et al., 2024). Generative and agentic AI systems are increasingly being used for learning and development within organizations (Dutta and Kannan Poyil, 2025). Agentic AI offers new approaches to training through on‑farm delivery, personalization, and real-time coaching, with automated assessment and integration into existing learning systems (Kostopoulos et al., 2025; Liu et al., 2025). Artificial intelligence systems can also easily adapt to changing demographics and different languages in multicultural dairy workplaces (Durst et al., 2018; Collins and Bayliss, 2020). Learning plans could be easily adapted to fit the pace of the learner and adapt if they are struggling. Learning assessment and verification can be conducted by agents, verifying skill mastery (Kostopoulos et al., 2025).

Farming practices are supported by an extensive research and knowledge system. Key actors include extension services and rural professionals associated with finance, veterinary, fertilizer, farm equipment servicing, and commercial products sectors. The dairy farm advisory system has been evolving with increased digitalization (Eastwood et al., 2019a; Nettle et al., 2022) and agentic AI may reshape farmer knowledge networks. There is an opportunity for knowledge and advisory systems to evolve to capture benefits of agentic AI, such as creating L1 (decision-tree) AI workflows that are “rented out” (either free or on a subscription model) to farmers to support certain decisions or activities (e.g., feed budgeting, grazing, and reproductive decisions). Agentic systems that can access farm systems models and enable predictive analytics and creation of digital twins (Lee et al., 2025; Liu et al., 2025) could provide advisors with much greater capability to address complexity and uncertainty for farmers. Higher-order agents that are semi-autonomous could be used to both assess farm-specific requirements for products such as fertilizer and animal health and close the loop with procurement and stock reconciliation. Such uses of AI would add to the service offerings of advisors and potentially create a technology “lock-in” (Carolan, 2020) that creates a competitive advantage for service and technology providers. There may also be first-mover advantages, for as Bornet et al. (2025) note, AI agents can lead to “compounding intelligence advantages” (p. 23) due to increased learning and value resulting from use and feedback. In the science knowledge domain, the use of GenAI and agents is quickly changing how data are analyzed (Ghavi Hossein-Zadeh, 2025), how published knowledge is reviewed, and how science information is communicated (Liu et al., 2025). Agentic AI promises transformative change in the generation and dissemination of knowledge.

Digitalization of pasture-based farm management and wider supply chains has greatly expanded the data available for operational, tactical, and strategic decisions (Leddin et al., 2023; Romera et al., 2024). Increasing data availability presents farmers with a challenge around effective data utilization. Although there are established decision rules for management of pasture-based dairying (Roche et al., 2007; Macdonald et al., 2010; e.g., grazing management, animal health and reproduction, environmental practices), the increasing complexity, variability, and regulation present a risk of increased cognitive load for decision-makers (Edwards et al., 2024; Lei et al., 2024). Additionally, the difference between average and good practice can be related to small changes in accuracy and timing of decisions (Hall et al., 2019). Agentic AI will have a strong role to play in helping farmers manage future complexity by leveraging the increased digital data. It could also enable precision farming at finer scales than can be effectively achieved by farmers, for example, cow-level, hourly, or square-meter-level decisions. Autonomous systems can reduce cognitive load (Skulmowski and Rey, 2017) related to decision-making and data overload. Use of AI may be a double-edged sword, however, as farmers will face learning challenges around new tools and they will add an increased requirement for review and oversight.

Unlocking the potential of data from dairy farms has to date been limited by issues such as technology integration, data interoperability, data quality, and the ability of farmers and advisors to interrogate data (Bahlo et al., 2019; Eastwood et al., 2023). Agentic AI provides a potential step change in precision farm management through multimodal capabilities (He et al., 2025), potential to accommodate text, imagery, and audio as well as numeric data, and ability to both access and manipulate existing models and software (Ferreira and Dórea, 2025; Li et al., 2025). Unified and standardized communication standards are required to enable agent interoperability (Li et al., 2025). To realize the AI opportunity, there needs to be a focus on “AI-ready” farm data in terms of clean and accurate data, digitalization of data, consistent data formats, FAIR (findable, accessible, interoperable, reusable) data (Bahlo and Dahlhaus, 2021), farmer ownership of data, and good metadata for context (Sullivan et al., 2024; Cabrera et al., 2025).

Beyond the potential use cases outlined previously, there are broader considerations related to development, application, and governance of AI tools (Ryan, 2025). Agentic AI represents a paradigm shift, transforming ML tools into autonomous actors capable of perceiving, reasoning, and acting over extended time horizons (Russell and Norvig, 2020). In pasture-based dairying, such systems could monitor herds, adapt feeding or irrigation schedules, and coordinate logistics. Yet their autonomy introduces novel governance, data integrity, and socio-technical challenges. Ensuring alignment with farmer and societal values requires a re-orientation of the research and innovation (**R&I**) ecosystem toward adaptive, risk-aware governance and human-centered oversight (Ji et al., 2025).

Traditional compliance models are ill-suited to self-directed systems operating in open farm environments. Governance must become dynamic, balancing technical progress (“forward alignment”) with continuous assurance (“backward alignment”; Maslej et al., 2025). The Institute of Electrical and Electronics Engineers (IEEE) Ethically Aligned Design and the European Union AI Act articulate principles, human dignity, transparency, and accountability (Ryan and Stahl, 2021); that should now be embedded directly into agricultural innovation programs. Within the pastoral sector, these principles imply multi-stakeholder coordination among government agencies, agricultural technology firms, and farming cooperatives to co-develop safety and performance standards for autonomous farm agents. Lifecycle risk assessment, spanning model training, deployment, and on-farm operation should be mandatory, with clear accountability across stakeholders.

The reliability of agentic AI depends on the integrity and provenance of its training data. Audits show that more than 70% of datasets used in AI lack adequate license information, while nearly half are miscategorized (Maslej et al., 2025). Data quality and integration issues have also been identified as obstacles to AI use in the agrifood sector (Benfenati et al., 2025). Combined with rising web-data restrictions, ‘robots.txt’ exclusions (exclusion protocol accessed by web crawlers when visiting websites) increased from 10% (2017) to 48% (2024); this has produced a data-consent crisis (Longpre et al., 2024). Dairy sector R&I must therefore develop methods for AI learning under data scarcity and for federating distributed sources such as sensors, herd-management software, and regional climate databases. Transparent metadata and traceable datasets should become standard (Kalokyri et al., 2025).

Bias mitigation is equally critical (Afreen et al., 2025). Human annotation often embeds cultural or moral bias; R&I programs should explore creation of moral datasets encoding sector-specific ethical norms, for example, balancing animal welfare outcomes with productivity goals (Dawkins, 2025; Foris et al., 2025). Because agentic systems rely on persistent memory, secure architectures must implement integrity checks and access controls to prevent memory poisoning or unauthorized cross-agent information flow (Huang and Hughes, 2025; Sapkota et al., 2026). Autonomous does not have to mean unattended (Cajueiro and Celestino, 2026), particularly in domains like dairy farming, where contextual and tacit judgment is indispensable (Eastwood et al., 2012; Ingram and Maye, 2020). Oversight models can be stratified by risk: human-in-the-loop for high-stakes domains such as reproduction and biosecurity, human-on-the-loop for routine optimization (e.g., feed allocation), and human-in-command for system-level governance and ethical review.

Embedding meaningful human control requires agent design that augments rather than replaces farmer expertise (Eastwood et al., 2021). Explainability and interpretability tools are prerequisites for trust (Eastwood et al., 2012). Comprehensive audit trails should record prompts, responses, and decision points, forming the evidential basis for accountability and continuous learning (Königstorfer and Thalmann, 2022).

Decisions in pasture-based dairy differ widely in acceptable error margins. Small inaccuracies in estrus detection or mating timing can have multiseason economic impacts, whereas feed-mixing errors may be recoverable. The R&I therefore needs frameworks linking an agent's required accuracy to the user's risk tolerance (Huang and Hughes, 2025). Calibration can draw on self-reported, observed, or experimental measures of risk attitude (Holt and Laury, 2002). Potential risks associated with agentic AI specifically in pasture-based dairying include technically correct but contextually wrong decisions (e.g., choosing the wrong paddock to graze when wet weather is coming), decision-making that has adverse environmental or animal welfare impacts (e.g., poor management of riparian zones or detection of animal health issues), and social license concerns (e.g., perceptions of AI-driven farming that undermine the image of dairying) (Espinoza-Sandoval et al., 2024; Ryan and Blok, 2026).

Digitalization of farm operations introduces new attack surfaces and therefore a focus on cybersecurity in agentic AI development is required (Huang and Hughes, 2025). Crucially, farmer control of their data needs clear protocols and data-sovereignty clauses, and technical enforcement of access rights are prerequisites for trustworthy adoption.

Agentic AI will reshape agricultural labor, displacing routine monitoring roles while creating demand for data-literate farm managers, AI technicians, and ethics auditors (Bhat et al., 2025). Addressing the question “Will it take my job?” requires reframing AI as augmentation, not replacement (Ryan, 2025). Workforce transition strategies, upskilling, lifelong learning, and advisory-network training, should accompany deployment. Ethical-impact assessments across the AI lifecycle can anticipate distributional effects and ensure equity (Omotayo et al., 2025; Williamson and Hartley, 2025). Organizational cultures that reward value-based design and empower employees to raise ethical concerns will foster durable trust.

Pasture-based farms are complex, data-rich ecosystems. The greatest promise of agentic AI lies in integrating heterogeneous information streams into unified decision support, requiring standardized communication protocols between agents. Current frameworks such as model context protocols improve tool interoperability but not interagent reasoning; emerging research into cooperative multiagent communication and formal-verification methods (Abd Alrahman and Piterman, 2021) is directly relevant. Hybrid intelligence paradigms, where human expertise and AI inference co-evolve, offer a pathway to blend tacit farmer knowledge with ML outputs (Eastwood et al., 2012; Maslej et al., 2025). Edge AI will also be crucial to maintain privacy and resilience under rural connectivity constraints (Tariq et al., 2025).

Although agentic AI is promising, there is a current gap between hype and reality (Bornet et al., 2025). Practical deployment in pasture-based dairying will need to overcome challenges related to the dynamic biological and climatic environment, concerns over privacy and data ownership, and ability to integrate with existing databases and farm software systems. Transparency and explainability are vital for not only building farmer trust in agent actions and recommendations, but also to enable farmers to cross-check the AI decision process with their own tacit knowledge and provide feedback to the agent (Li et al., 2025). Transparency requires the open disclosure of data sources, algorithms, and performance metrics; however, this remains limited (Maslej et al., 2025). Improved explainability could help bridge gaps in farmer trust, for example, “explainable agents” can justify intended actions before execution, allowing farmers to cross-check and validate reasoning (Sapkota et al., 2026).

In the near term, pasture-based agents will remain task-specific, such as handling feed optimization or anomaly detection, but will evolve toward multiagent ecosystems coordinating full-farm operations and linking to climate-adaptive models (Omotayo et al., 2025; Tirulo et al., 2026). These systems promise productivity gains and cognitive-load reduction yet must progress together with assurance.

The development of agentic systems requires engagement of farmers and stakeholders in the ideation and prototyping stages. Farmer-centered design processes have been applied in technology development in the past decade (Eastwood et al., 2022; Lee et al., 2025). These design processes can help developers and farmers co-explore aspects of responsible innovation (Ingram et al., 2022; Romera et al., 2024) to address issues of trust, explainability, transparency, ownership of data created by AI, and privacy (Omotayo et al., 2025). Research is needed on how farmers engage, interpret, and trust AI outputs, and how farmers react if AI decision-making conflicts with their own intuition or decisions, or if the AI output is incorrect. The anticipation of unintended consequences and potential harm is a key part of this design process (Chan et al., 2023). Building on research in human-AI collaboration (Acharya et al., 2025), farmer-AI interaction studies are warranted to address some of these issues, along with research focused on hybrid intelligence where the knowledge of farmers and agentic AI interacts in decision processes.

Agentic AI development remains uncertain. Current activity focuses on simple agents that address specific topics (Lamanna et al., 2025; Liu et al., 2025). In the medium term, the development of multiagent systems will add more system-level value to farmers. Fully agentic systems that can reason and self-correct, such as climate-adaptive agents, offer the most promise, but the timeline to realization of such systems relies on technical, data engineering, and social factors (Nisa et al., 2026). Sectors that are at the frontier of agentic AI adoption include healthcare, finance, manufacturing, and information technology (Acharya et al., 2025). For the pasture-based dairy sector to be a fast follower requires focused R&I effort, understanding of farmer-AI interactions and opportunities, and addressing the major socio-technical challenges.

This review identifies key R&I needs for development of pasture-based dairy agentic AI. A priority is ensuring AI-ready data. A focus on data will enable more effective AI utilization, and requires ongoing progress on digitalization, interoperability, open data standards, data quality, data ownership, and sovereignty (Acharya et al., 2025; Omotayo et al., 2025). Farmer trust will be a primary barrier for adoption, necessitating greater research into incorporating explainability and transparency in dairy AI (Ghavi Hossein-Zadeh, 2025) along with education initiatives and training to improve farmer understanding and confidence in AI. To direct R&I effort requires needs-analysis research to identify most valuable use cases for agentic AI. Farmer-AI interaction studies should explore processes for building farmer understanding, capability, and trust. Additionally, further understanding of the required dairy workforce implications and reskilling strategies is required. Hughes et al. (2025) note the promise of agentic AI requires “robust governance frameworks, cross-industry collaboration, and interdisciplinary research into ethical design” (p. 489). Legislative barriers and possible legislative changes need to be assessed with respect to responsible and effective AI use (Espinoza-Sandoval et al., 2024).

Agentic AI represents a paradigm shift in pasture-based dairy farming, offering the potential to enhance decision-making, productivity, and sustainability through autonomous, data-driven systems. Its successful integration depends not only on technical capability but also on trust, transparency, and alignment with farmer values. By embedding human-centered design, ethical governance, and hybrid intelligence approaches, agentic AI can support, rather than replace, farmer expertise. Regardless of the use cases and agentic AI development trajectory in dairy farming, collaborative R&I will be essential to ensure that agentic systems deliver meaningful benefits while safeguarding the integrity of pasture-based farming communities.
